# The monoclonal antibody Zt/f2 targeting RON receptor tyrosine kinase as potential therapeutics against tumor growth-mediated by colon cancer cells

**DOI:** 10.1186/1476-4598-10-82

**Published:** 2011-07-12

**Authors:** Hang-Ping Yao, Yong-Qing Zhou, Qi Ma, Sunny Guin, Snehal S Padhye, Rui-Wen Zhang, Ming-Hai Wang

**Affiliations:** 1 State Key Laboratory for Diagnosis and Treatment of Infectious Diseases, First Affiliated Hospital, Zhejiang University College of Medicine, Hangzhou, Zhejiang 310003, P. R. China; 2 Division of Neurosurgery, First Affiliated Hospital, Zhejiang University College of Medicine, Hangzhou, Zhejiang 310003, P. R. China; 3 Department of Biomedical Sciences and Cancer Biology Center, School of Pharmacy, Texas Tech University Health Sciences Center, Amarillo, Texas 79106, USA; 4 Department of Pharmaceutical Sciences, School of Pharmacy, Texas Tech University Health Sciences Center, Amarillo, Texas 79106, USA

## Abstract

**Background:**

Overexpression of the RON receptor tyrosine kinase contributes to epithelial cell transformation, malignant progression, and acquired drug resistance. RON also has been considered as a potential target for therapeutic intervention. This study determines biochemical features and inhibitory activity of a mouse monoclonal antibody (mAb) Zt/f2 in experimental cancer therapy.

**Results:**

Zt/f2 is a mouse IgG2a mAb that is highly specific and sensitive to human RON and its oncogenic variants such as RON160 (ED_50 _= 2.3 nmol/L). Receptor binding studies revealed that Zt/f2 interacts with an epitope(s) located in a 49 amino acid sequence coded by exon 11 in the RON β-chain extracellular sequences. This sequence is critical in regulating RON maturation and phosphorylation. Zt/f2 did not compete with ligand macrophage-stimulating protein for binding to RON; however, its engagement effectively induced RON internalization, which diminishes RON expression and impairs downstream signaling activation. These biochemical features provide the cellular basis for the use of Zt/f2 to inhibit tumor growth in animal model. Repeated administration of Zt/f2 as a single agent into Balb/c mice results in partial inhibition of tumor growth caused by transformed NIH-3T3 cells expressing oncogenic RON160. Colon cancer HT-29 cell-mediated tumor growth in athymic nude mice also was attenuated following Zt/f2 treatment. In both cases, ~50% inhibition of tumor growth as measured by tumor volume was achieved. Moreover, Zt/f2 in combination with 5-fluorouracil showed an enhanced inhibition effect of ~80% on HT-29 cell-mediated tumor growth *in vivo*.

**Conclusions:**

Zt/f2 is a potential therapeutic mAb capable of inhibiting RON-mediated oncogenesis by colon cancer cells in animal models. The inhibitory effect of Zt/f2 *in vivo *in combination with chemoagent 5-fluorouracil could represent a novel strategy for future colon cancer therapy.

## Background

The RON (recepteur d'origine nantais) protein belongs to the MET proto-oncogene family [1], which constitutes a unique subfamily of receptor tyrosine kinases [2]. Roles of RON in tumor progression have been studied in both *in vitro *and *in vivo *models [3]. RON is overexpressed in various types of primary tumor samples including colon, breast, and pancreatic cancers [4-7]. In colon and breast cancers, RON overexpression associates with the diseases at any stage and serve as an independent predictor of subsequent relapse [6-8]. Transgenic studies show that RON overexpression in lung and mammalian tissue causes tumor formation and promotes tumor metastasis [9-11]. Biochemically, RON overexpression results in constitutive tyrosine phosphorylation, which stimulates downstream signaling cascades including RAS-MAP kinase and PI-3 kinase-AKT pathways [3,12]. These activities lead to cell morphological changes with increased cell invasive activity [13,14]. Clearly, altered RON expression is a tumorigenic factor contributing to malignant phenotypes of epithelial cancers.

RON is a 180 kDa heterodimeric protein composed of a 40 kDa extracellular α-chain and a 150 kDa transmembrane β-chain with intrinsic tyrosine kinase activity [1]. RON is recognized and activated by a ligand known as macrophage-stimulating protein (MSP) [15,16], also known as hepatocyte growth factor-like protein [17]. The binding of MSP to RON extracellular sequences causes receptor dimerization, which leads to auto-phosphorylation of tyrosine residues in the intracellular sequences, creates the docking motifs for interaction with signaling molecules, and subsequently increases the tyrosine kinase activity [18]. The RON extracellular sequences contain several functional motifs including a sema domain followed by a cysteine-rich hinge (PSI), three immunoglobulin-plexin-transcription (IPT) units, and a peptide of 97 amino acids previously thought to contain the 4^th ^IPT unit [1]. The sema domain stretches in both α and β chains and is known to contain high affinity binding site for MSP [19,20]. The exact role of PSI is unknown. PSI seems to act as a link that regulates receptor conformation upon MSP binding to RON [18]. The IPT units are important in RON activity. Elimination of the first IPT domain coded by exons 5 and 6 results in the formation of a RON variant known as RON160, which possesses oncogenic activity [21]. Functions of the second and third IPT units are currently unknown. A 97 amino acid peptide (from Pro^861 ^to Thr^957^) stretches between the last amino acid Leu^860 ^of the 3^rd ^IPT and the first amino acid Leu^958 ^of the transmembrane segment [22]. Forty-nine amino acids (from Tyr^884 ^to Gln^930^) in this sequence are coded by exon 11, which often is deleted through the splicing process [23,24]. This deletion results in formation of a single-chain precursor RON165, which is retained in cytoplasm [23,24]. Exon 11 deletion also causes spontaneous RON dimerization and phosphorylation [23,24]. Thus, the sequences encoded by Exon 11 are critical in RON maturation and activation process (referred as maturation-required sequences, MRS). Considering the importance of extracellular domains in ligand binding, receptor maturation, and activation, it is believed that biological or chemical agents that specifically interact with these domains should regulate RON activation and control its downstream signaling events. Such studies should also provide a basis for the development of potential therapeutics designed to inhibit RON-mediated tumorigenesis.

Pathogenesis of RON in epithelial cancer has made this receptor an attractive drug target [25-27]. Potential therapeutics including small molecule kinase inhibitors (SMI), mAbs and small interfering (si) RNA have been developed and tested to block RON-mediated tumorigenesis [14,25-28]. Results from these studies demonstrate that blocking RON signals contributes to reduced cell growth, diminished cell invasiveness, and impaired tumor metastasis. Studies from *in vivo *models further demonstrate that SMI and mAb specific to RON inhibit tumor growth in various xenograft models [25,26]. Thus, RON is a drug-targeting candidate, which has potential to be used clinically in targeted cancer therapy. The present work determines the biological features of a novel mAb specific to MRS in RON extracellular sequences. Biochemical analysis indicates that the interaction of the mAb with MRS facilitates RON internalization followed by degradation. Moreover, administration of this mAb as a single agent partially inhibits xenograft tumor growth *in vivo *and potentiates the cytotoxic effects of chemotherapeutics.

## Results

### Biochemical characterization of mAb Zt/f2 specific to human RON

**Figure 1:**
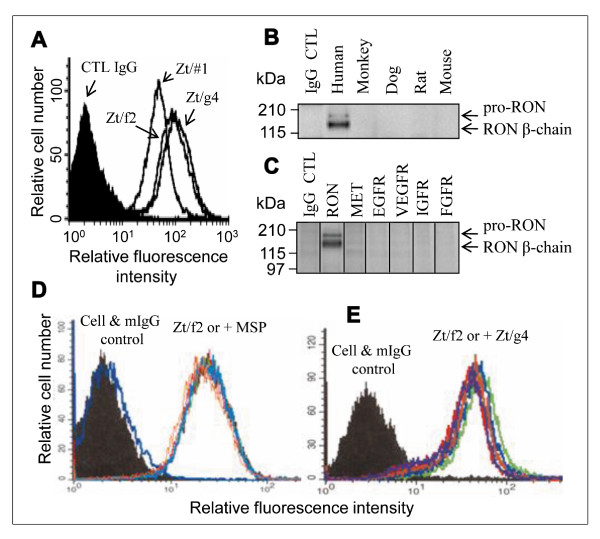
**Mouse mAb Zt/f2 is highly specific and sensitive to human RON: **A**) Zt/f2 recognizes an epitope on RON extracellular sequences**. 3T3-RON cells (0.5 × 10^6 ^cells per sample) were incubated at 4°C for 45 min with 1 μg/ml of Zt/f2, Zt/#1, Zt/g4, or control mouse IgG, respectively. Goat anti-mouse IgG coupled with FITC were used as the detecting antibody followed by flow cytometry analysis. **B**) Zt/f2 does not recognize RON homologues from different species. Cellular proteins (300 μg per sample) from cell lines known to express RON homologues were incubated with Zt/f2 (10 μg per sample) overnight followed by addition of Protein G-Sepharose. Immunoprecipitated proteins were separated in 8% SDS-PAGE under reduced conditions. Rabbit IgG cross-reacting with RON homologues (Santa Crutz Biotechnology) from different species (50 μg cellular protein/sample from cell lines: human HCT116, monkey COS-1, canine MDCK, rat IEC18, and mouse peritoneal macrophages) were used followed by ECL reactions. **C**) Zt/f2 only recognizes RON but not other RTKs. Cellular proteins (300 μg per sample) from cell lines known to express MET (HCC1937), EGFR (BxPC3), VEGFR (HT-29), IGFR (AsPC-1), and FGFR (MDA-MB-361) were incubated with Zt/f2 (1 μg per sample) followed by immunoprecipitation with Protein G-Sepharose. Western blot analysis was performed as in **B **using specific antibodies. **D**) &**E**) Zt/f2 does not compete with MSP or Zt/g4 for RON. 3T3-RON cells were incubated with 3 nM of FITC-labeled Zt/f2 alone or with increased amounts of MSP (**D**) at 0, 5, 10, 20, 40, 60 nM, or Zt/g4 (**E**) at 0, 10, 25, 50, 100, and 160 nM, respectively. The fluorescent intensity of each sample was plotted in individual figures.

Anti-RON mAbs were obtained by classical hybridoma methods [4,29] and characterized (Table 1). Zt/f2 was selected for further evaluation. Immunofluorescent analysis indicated that Zt/f2 binds to an epitope(s) on RON extracellular sequences (Figure 1A) and displays similar binding affinity as Zt/g4 at a defined concentration. Zt/f2 recognized the native RON receptor but not denatured protein under reduced conditions (data not shown). Species cross-reactivity studies showed that Zt/f2 is highly specific to human RON and does not recognize RON homologues expressed by monkey, dog, rat, and mouse (Figure 1B). Immunoprecipitation analysis of the cross-reactivity with different RTK proteins showed that Zt/f2 at 10 μg per ml fails to recognize MET, EGFR, IGFR, FGFR, and VEGFR (Figure 1C). To determine if Zt/f2 competes with MSP or Zt/g4 for binding to RON, we performed binding competition experiments. Results in Figure 1D showed that the binding of 3 nM FITC-labeled Zt/f2 to 3T3-RON cells was not inhibited by increased amounts of MSP. The fluorescence intensity from individual samples containing MSP ranging from 1 to 40 nM was all overlaid with the sample containing Zt/f2 alone. Similarly, the Zt/f2 binding to RON was not replaced by the increased amounts of Zt/g4 (Figure 1E), which recognizes RON sema domain [29]. Taken together, results in Figure 1 demonstrate that mAb Zt/f2 is highly specific and sensitive to human RON. It binds to an epitope(s) on the RON extracellular sequences, which differs from MSP and Zt/g4.

**Table 1:** Biochemical and Biological Features of Mouse Anti-RON Monoclonal Antibodies in Various Applications*

Features	Zt/f2	Zt/#1	Zt/g9
**IgG subtype**	IgG2a	IgG1	IgG1
**Specificity**	Only human RON	Only human RON	Only human RON
**Recognition**	MRS	Sema	Sema
**ED_50_**	2.3 nmol/L	3.7 nmol/L	4.2 nmol/L
**Reactivity to** **Isoforms**	RON++++ RON160++++ RON165^_^ RON155^_^ RON110+++	RON+++, RON160++++, RON165+++, RON155++ RON110^_^	RON+++, RON160+++ RON165+++, RON155++ RON110^_^
**Cross-reactivity with MET and EGFR**	Negative	Negative	Negative
**Induction of phosphorylation**	+++	++	ND
**Western blotting**	Negative	Negative	Negative
**Immunoprecipitation**	++++	+++	++++
**Cell surface immunofluorescence**	++++	+++	+++
**Immunohistochemical staining**	++++	++	+++
**Effect on RON expression**	Down-regulation	Down-regulation	ND

*The reactivity of Zt/f2, Zt/#1, and Zt/g9 in various immune assays were determined as detailed in Materials and Methods. For immunofluorescence and immunoprecipitation, individual mAbs were used at 1 μg/ml per sample. In immunohistochemical staining, mAbs were used at 2 μg/ml. Experiments were repeated twice. The reactivity/intensity indicated by + or-was obtained by comparing to anti-RON mAb Zt/g4 [29]. MRS, maturation-required sequences; ND, not done.

### Recognition by Zt/f2 of MRS in the RON extracellular domains

**Figure 2:**
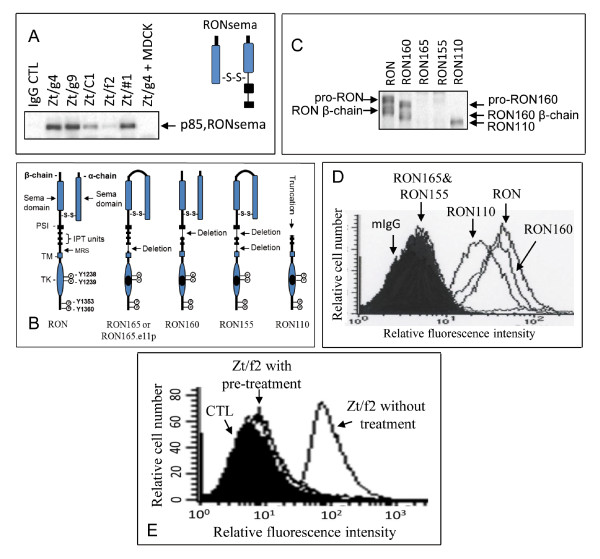
**Zt/f2 binds specifically to MRS in the RON β-chain extracellular sequence**. **A**) Zt/f2 does not bind RONsema. Recombinant RONsema (0.5 μg per sample) from transfected cells (Ma et al., 2010) was immunoprecipitated with 2 μg/ml Zt/f2 or other mAbs with Protein G-Sepharose. Samples were analyzed under non-reduced conditions by Western blot analysis using antibodies to RON extracellular sequences (Wang et al., 1994). **B**) Schematic representation of RON and its variants. General structures of RON are illustrated on the left. The α- and β-chains are indicated. Deleted regions in individual variants are marked with arrows. TM, transmembrane segment; MRS, maturation-related sequences; and TK, tyrosine kinase domain. **C**) Zt/f2 does not interact with RON165 and RON155. Cellular proteins (300 μg per sample) from NIH3T3 cells expressing RON, RON160, RON165, RON155, or RON110 were immunoprecipitated with 2 μg/ml of Zt/f2. Samples were detected by Western blot analysis using rabbit IgG antibody to RON C-terminal peptide (Xu et al., 2004). **D**) Zt/f2 does not bind to RON165 and RON155 in intact cells. NIH3T3 cells expressing individual RON variants (0.5 × 10^6 ^cells per sample) were permeabilized as previously described (Lu et al., 2007) and then incubated with 2 μg/ml of Zt/f2. Fluorescence was detected by FITC-coupled goat anti-mouse IgG. **E**) MRS peptide blocks Zt/f2 binding to RON. Zt/f2 (2 μg/ml) was first mixed with or without 1 μM of synthetic peptide for 30 min and then incubated with 3T3-RON cells. Cell surface fluorescence was determined as described above.

To determine the potential binding region of Zt/f2, we first tested if Zt/f2 binds to a naturally occurring RON variant (known as RONsema) comprising the entire sema domain, PSI hinge, and a N-terminal portion of the first IPT unit [20]. Results from co-immunoprecipitation showed that control mAb Zt/g4 and Zt/#1 strongly interacted with the RONsema (Figure 2A), indicating that these mAbs bind to an epitope(s) located on the RONsema proteins. However, incubation of Zt/f2 with RONsema did not result in any detectable RONsema in Western blot analysis after co-immunoprecipitation (Figure 2A), suggesting that the binding region of Zt/f2 is not located on the RONsema protein.

The interaction of Zt/f2 with RON160, RON165, RON155, and RON110 was then tested with lysates from NIH-3T3 cells expressing individual RON variants [30]. These variants have deletion in the first IPT unit (RON160), MRS (RON165), the first IPT plus MRS (RON155), and truncation at the first IPT unit (RON110) (Figure 2B) [30]. Results in Figure 2C showed that Zt/f2 recognizes RON, RON160, and RON110, all of which contain MRS. However, Zt/f2 did not recognize RON155 and RON165, since both lack MRS. Immunofluorescence analysis of Zt/f2 binding to 3T3 cells expressing individual RON variants confirmed these results (Figure 2D). Zt/f2 binds to RON and RON160 with comparable affinity. The affinity of Zt/f2 binding to RON110 was moderate. However, Zt/f2 failed to bind to RON165 or RON155. To confirm if MRS was involved, Zt/f2 was pre-incubated with a 49 amino acid peptide coded by exon 11 followed by fluorescence cell surface analysis. Results in Figure 2E show that after pre-incubation, Zt/f2 almost completely lost the ability to bind to RON. Similar results were also seen in 3T3-RON160 cells (data not shown). Taken together, results in Figure 2 demonstrate that Zt/f2 recognizes an epitope(s) located in MRS coded by exon 11 in the RON β-chain extracellular sequences.

### Effect of Zt/f2 on RON phosphorylation and downstream signaling cascades

**Figure 3:**
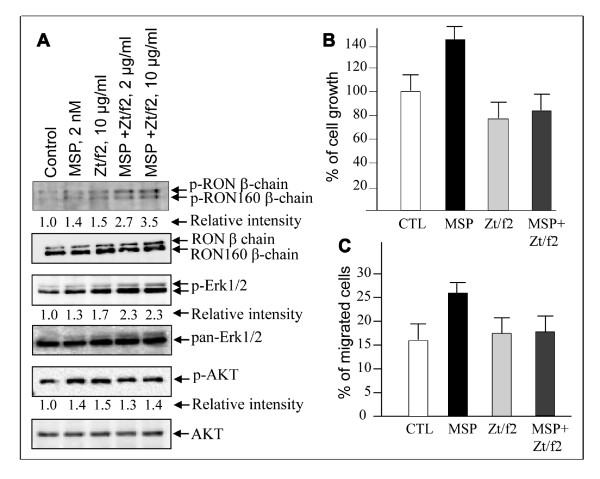
**Effect of Zt/f2 on RON/RON160 phosphorylation, cell proliferation, and migration by HT-29 cells**. **A) **Serum-starved HT-29 cells (3 × 10^6 ^cells/sample) were stimulated for 15 min at 37°C by 2 nM MSP, 10 μg/ml Zt/g4, or both followed by immunoprecipitation using Zt/g4. Cells treated with control IgG served as the control. Phospho-RON/RON160 was determined by Western blot analysis using anti-PY100 mAb. Phosphorylation of Erk1/2 and AKT were determined by Western blot analysis using specific antibodies. **B**) HT-29 cells in wells of a 96-well plate (10,000 cells/well) were treated with MSP (2 nM), Zt/f2 (10 μg/ml), or both in triplicate for three days. Cells numbers were counted as detailed in Materials and Methods. **C**) A 48-well cell migration chamber was used to determine levels of HT-29 cell migration in a period of 24 h. The bottom wells of the chamber were filled with MSP (2 nM), Zt/f2 (10 μg/ml), or both. The top wells were filled with HT-29 cells (1 × 10^4 ^cells/well) in DMEM with 5% FBS. Cells migrated and attached to the membrane were counted as previously described (Wang et al., 1994).

Deletion of MRS results in constitutive phosphorylation of RON [23,24]. To determine if Zt/f2 binding to this region affects RON activation, cells were serum-starved overnight and treated with MSP or Zt/f2 for 15 min. A trace of RON and RON160 phosphorylation was detected in HT-29 cells (Figure 3A). MSP stimulation slightly increased the levels of phosphorylation. Zt/f2 treatment alone results in a slight increase in RON phosphorylation. A moderate level of synergism in RON phosphorylation was observed when Zt/f2 was combined with MSP. Also, Zt/f2 alone moderately increased Erk1/2 and AKT phosphorylation. We observed a moderate increase in Erk1/2 or AKT phosphorylation when MSP and Zt/f2 were combined. To study if phosphorylation translates into cell proliferation, the effect of Zt/f2 on HT-29 cell growth was determined. Treatment of cells with Zt/f2 for three days moderately inhibited cell growth compared to control or MSP-stimulated cells (Figure 3B). Similarly, Zt/f2 moderately inhibited MSP-induced cell proliferation. Analysis of cell transmembrane migration revealed that Zt/f2 only has marginal effect on HT-29 cell migration (Figure 3C). A moderate inhibition by Zt/f2 in MSP-induced cell migration also was observed. Taken together, results in Figure 3 suggest that although Zt/f2 slightly increases RON, Erk1/2, and AKT phosphorylation, it moderately inhibits MSP-stimulated or non-stimulated HT-29 cell growth but only slightly prevents these cell migrations.

### Down-regulation by Zt/f2 of RON and its variant expression by various cancer cells

**Figure 4:**
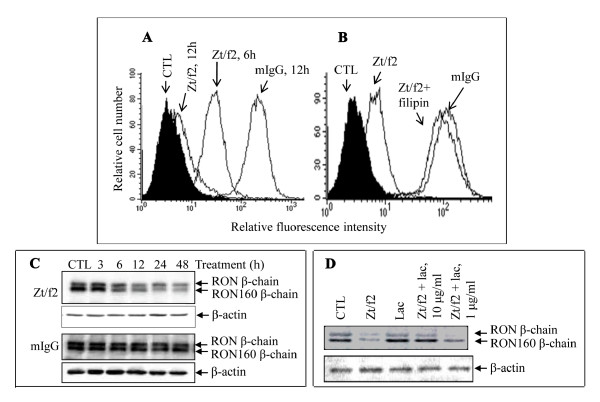
**Effect of Zt/f2 on RON expression by colon cancer cells**. **A**) Down-regulation of RON/RON160 expression by Zt/f2. HT-29 cells (1 × 10^6 ^cells/sample) were treated with Zt/f2 (10 μg/ml) or control mouse IgG at 37°C for 6 and 12 h. After treatment, cells were washed with acidic buffer to eliminate surface-bound antibody (Guin et al., 2010) and then incubated with Zt/g4 (1 μg/sample) to detect RON/RON160 remaining on the cell surface by flow cytometric analysis. **B**) Preventive effect of filipin on Zt/f2-induced RON reduction. HT-29 cells were pre-incubated with filipin (10 μg/ml) for 30 min and then treated at 37°C for 12 h with Zt/f2 or control mouse IgG (10 μg/ml). After washing with acidic buffer, RON on cell surface was detected by Zt/g4 as previously described (Guin et al., 2010). **C**) Kinetic effect of Zt/f2 on RON/RON160 expression. HT-29 cells were treated with 10 μg/ml of Zt/f2 for various times. RON expression was determined by Western blot analysis. Normal mouse IgG was used as the control. **D**) Preventive effect of lactacystin on Zt/f2-induced RON reduction. HT-29 cells were pre-incubated with 1 or 10 μg/ml of lactacystin for 30 min and then treated with 10 μg/ml of Zt/f2 at 37°C for 12 h. Levels of RON were determined by Western blot analysis.

To determine how Zt/f2 inhibits HT-29 cell growth, we studied the effect of Zt/f2 on RON/RON160 expression by HT-29 cells. After cells were treated with Zt/f2 for various time periods, levels of RON/RON160 expression were determined by cell surface fluorescence analysis. Results in Figure 4A showed that the binding of Zt/f2 to HT-29 cells at 37°C caused the down-regulation of the cell surface RON/RON160 expression. More than 80% of cell surface fluorescence was reduced after Zt/f2 incubation for 12 h. The down-regulation seemed to be linked to cellular endocytosis because fluorescence intensity was significantly recovered when endocytic inhibitor filipin was used (Figure 4B) [31]. Filipin alone had no effect on RON expression (data not shown); however, when used with Zt/f2, Zt/f2-induced down-regulation was significantly prevented. Western blot analysis of RON/RON160 expression further confirmed that Zt/f2 treatment results in a significant RON/RON160 reduction (Figure 4C). Consistent with cell surface fluorescence analysis, both RON and RON160 expression were progressively reduced up to 48 h after Zt/f2 treatment. In control cells treated with normal mouse IgG, the levels of RON expression were maintained. The diminished RON expression seems to be caused by intracellular proteasomic degradation. Pretreatment of HT-29 cells with proteasomic inhibitor lactacystin significantly prevented Zt/f2-induced RON/RON160 reduction (Figure 4D). More than 90% of RON/RON160 was recovered from cells treated with Zt/f2 plus 10 μg/ml of lactacystin. Thus, results in Figure 4 demonstrate that Zt/f2 treatment causes down-regulation of RON/RON160 expression by HT-29 cells. The reduced cell-surface RON/RON160 expression is mediated by endocytosis and subsequent degradation through proteasomic mechanism. The down-regulation could lead to Zt/f2-induced reduction in cell growth and migration seen in Figure 3.

### Inhibitory effect of Zt/f2 *in vivo *on tumor growth mediated by 3T3-RON160 or colon cancer cells

**Figure 5:**
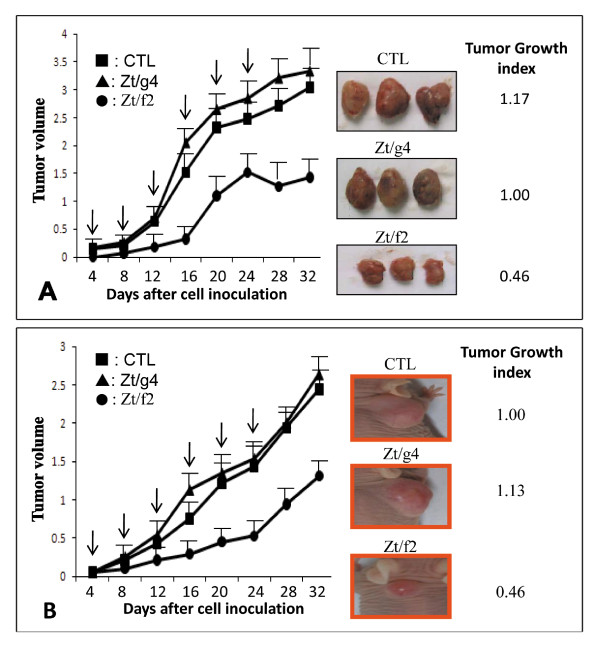
**Inhibitory effect of Zt/f2 on 3H3-RON160 or HT-29 cell-mediated tumor growths**. Induction of tumors in Balb/c mice by NIH3T3-RON160 cells (**A**) or in athymic nude mice by HT-29 cells (**B**) was detailed in Materials and methods. Tumor bearing mice were randomized into different groups (3 mice per group). Treatment of animals with Zt/f2, Zt/g4, or control mouse IgG at the defined dose and schedule as detailed in Materials and Methods. Mice were euthanized at day 32 in both experiments. The therapeutic effect of Zt/f2 on tumor growth was obtained by comparing tumor volumes among groups. Antibody injection is marked with arrows. Tumor growth index from control mice was set at 1.00 and used to compare the differences among groups.

The effect of Zt/f2 on 3T3-RON160-mediated tumor growth is shown in Figure 5. In this model, Zt/f2 treatment was delayed three days after cell inoculation. Tumor size increased in a time dependent manner (growth index as 1.00 at day 32) in the control group. An interesting finding was that Zt/g4 treatment slightly increased tumor growth mediated by 3T3-RON160 cells. In this case, a slight increase in tumor volume was observed. In contrast, Zt/f2 administration resulted in inhibition of tumor growth, an effect that was seen as early as five days after Zt/f2 initial injection. An average of 54% reduction in the tumor volume was observed as compared to tumors from control mice.

The therapeutic effect of Zt/f2 also was validated on colon HT-29 cell-mediated tumor model in athymic nude mice. Experimental procedures were similar to those described above. Repeated administration of Zt/f2 had an inhibitory effect on HT-29 tumor cell growth (Figure 5B). An average of 56% reduction in tumor volume was observed. Interestingly, Zt/g4 did not show the significant agonistic effect on HT-29 cell-induced tumor growth. These results, together with those from Figure 5A, demonstrate that Zt/f2 treatment partially inhibits tumor growth mediated not only by RON160 transformed mouse fibroblast cells, but also by colon HT-29 cancer cells that naturally express RON and RON160.

### Effect of Ztf2 with 5-FU on tumor growth in nude mice

**Figure 6:**
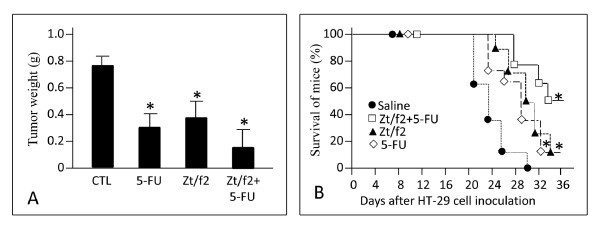
**Enhanced activities of Zt/f2 and 5FU in inhibition of tumor growth**. HT-29 cell-mediated tumor growth in athymic nude mice was used as the model (O'Toole et al., 2006). Zt/f2 treatment began when tumor volumes reached about 9 mm^3 ^(around 8 days after cell inoculation). **A**) Comparison of tumor weights from different groups of G-I. **B**) Survival rates in different groups of G-II. In both G-I and G-II, statistical significance (p < 0.05) is indicated with a star.

Results from above studies prompted us to study the combination of Zt/f2 and 5-fluoracil (5-FU) in tumor therapy. Tumor weight at the end of treatment was used as the evaluation marker. Results in Figure 6A show the inhibitory effect of Zt/f2, 5-FU, and combined treatment on HT-29 tumor growth in athymic nude mice. Treatment with 5-FU alone showed 59% decrease in tumor growth when compared with saline-treated mice. Administration of Zt/f2 as a single agent also caused 51% decrease in tumor weight. However, the combined treatment (Zt/f2 and 5-FU) showed maximal inhibition with a decrease of 80% in tumor weight. Furthermore, comparative survival rate results among different groups confirmed the effectiveness of the combined treatment (Figure 6B). All eight mice in the saline group died within 30 days due to complication of tumor growth. Zt/f2 or 5-FU treatment showed significant effect on prevention of animal death, and their survival rates significantly improved. In mice treated with Zt/f2 and 5-FU, four mice (50%) were still alive after a period of 36 days. Thus, results in Figure 6 demonstrate that Zt/f2 in combination with 5-FU prolongs the survival rate of tumor-bearing mice.

## Discussion

The major finding in this study is the therapeutic effectiveness of mAb Zt/f2 on tumor growth mediated by colon HT-29 and transformed 3T3 cells. RON as a drug target for potential cancer therapy has been under intensive investigation [25,26,32]. Various approaches including specific siRNA, mAb, and small molecule kinase inhibitors have been studied [25,26,32]. The data from current studies indicate that blocking RON signal has profound impact on tumor growth in animal models, which could have clinical implication in the treatment of human cancers. This conclusion is supported by data from analysis of Zt/f2 biochemical properties and by evaluation of the effect of Zt/f2 on tumor growth *in vivo*. Zt/f2 binds specifically to an epitope(s) located in MRS coded by exon 11 in the RON β-chain extracellular sequences. Although the binding of Zt/f2 results in RON phosphorylation, it subsequently causes RON internalization leading to diminished RON expression in cancer cells. Such effect attenuates RON transduced signals required for tumorigenic activities. As *in vivo *studies have shown, administration of Zt/f2 as a single agent partially inhibits tumor growth mediated by transformed 3T3 cells expressing RON160 in Balb/c mice and by colon HT-29 cells expressing RON and RON160. Moreover, enhanced effects were achieved when Zt/f2 was combined with chemotherapeutic agents such as 5-FU. Thus, by targeting RON/RON160 overexpressed by cancer cells such as colon cancer cells, Zt/f2 is capable of inhibiting tumor growth mediated by RON/RON160 signaling.

Zt/f2 specifically binds to an epitope in MRS coded by exon 11 in the RON β-chain extracellular sequences. This conclusion is derived by analyzing variants with different deletions or truncations in RON extracellular sequences and by competing with the synthetic peptide with known sequences. Zt/f2 does not recognize RONsema, a truncated RON protein containing the entire sema, PSI and a portion of the 1^st ^IPT unit [20]. However, Zt/f2 binds to RON160, which lacks the entire 1^st ^IPT domain [33]. Since the RON extracellular sequences contain more than 950 amino acids, analysis of Zt/f2 binding to RONsema and RON160 helps us investigate potential regions in the remaining β-chain extracellular sequences, which contain the 2^nd^, and 3^rd ^IPT units and the MRS. An important clue comes from the inability of Zt/f2 to bind to RON165, which has a deletion of MRS 49 amino acids between the 3^rd ^IPT and the transmembrane segment. The 49 amino acids are exclusively coded by exon 11 [22]. We also observed the failure of Zt/f2 to bind to RON155. RON155 has two deletions, one in 1^st ^IPT unit and the other in MRS. These results suggest that MRS could be the potential region that Zt/f2 binds. With the use of the synthetic peptide with 49 amino acids sequenced from exon 11, we showed that pre-incubation of Zt/f2 with MRS peptide almost completely prevents Zt/f2 binding to RON or RON160 expressed on the cell surface. Thus, Zt/f2 binds to an epitope that resides in MRS.

Zt/f2 binding to RON slightly increases receptor phosphorylation as shown Figure 3. Zt/f2 also moderately synergizes with MSP in enhancement of RON and Erk1/2 phosphorylation. These results suggest that Zt/f2 engagement with MRS is sufficient to cause conformational changes for dimerization leading to RON phosphorylation and activation of the downstream signaling cascade. However, Zt/f2 showed certain inhibitory effects on HT-29 cell growth and migration. These suggest a disconnection between Zt/f2-induced signaling events and biological outcomes, which partially resemble anti-EGFR mAb cetuximab and matuzumab. Cetuximab and matuzumab induce EGFR dimerization and phosphorylation, but fail to trigger downstream signaling by AKT and Erk. By inhibiting the EGF-induced activation of AKT and Erk, both mAbs inhibit lung tumor growth *in vivo *[34,35]. Currently, the mechanisms underlying disconnections between intracellular signaling and cellular activity are unknown. A possible explanation is that Zt/f2-induced signaling is not sustained long enough to initiate cell proliferation and migration activities. Down-regulation by Zt/f2 of RON expression as described in Figure 4 could be a major reason. We also have observed that phosphorylation of RON and Erk1/2 by HT-29 cells was completely absent after Zt/f2 treatment for 24 or 72 h (our unpublished data). Regardless of these observations, it will be interesting in the future to dissect cellular mechanisms responsible for such disconnections.

The findings that Zt/f2 induces RON internalization and degradation are interesting in terms of inhibitory effect *in vivo*. Down-regulation of RTK expression is an anti-tumor mechanism of therapeutic anti-RTK mAb such as trastuzumab specific to HER2 [36-38]. Results from current studies demonstrate that Zt/f2 treatment transiently induces RON phosphorylation followed by down-regulation of RON expression by HT-29 cancer cells. As shown in Figure 4A, levels of RON on cell surface were progressively diminished 6 h after Zt/f2 treatment. The mAb-induced RTK down-regulation is a complicated process involving receptor internalization followed by protein degradation through intracellular mechanisms [37,39]. We observed that the addition of filipin, an inhibitor of cellular internalization process [31], prevents the effect of Zt/f2 on RON internalization. These results suggest that Zt/f2-induced RON internalization is required for down-regulation. Moreover, proteasomal inhibitor lactacystin blocked Zt/f2-induced RON reduction. These data indicate that the internalized RON proteins are degraded through proteasomic mechanism. The diminished RON expression could affect tumorigenic activities of colon cancer cells leading to impairment of their growth *in vivo*.

Inhibition of HT-29 cell-mediated tumor growth provides direct evidence indicating that Zt/f2 alone or in combination with chemotherapeutic agent 5-FU has potential therapeutic activities *in vivo *against tumor cells expressing RON or RON160. Analysis of *in vivo *results revealed several features of Zt/f2 as a therapeutic agent. First, Zt/f2 used as a single agent is able to inhibit tumor growth mediated by HT-29 and RON160 transformed 3T3 cells. Repeated administration of mAb is required to achieve this effect. Second, Zt/f2 used in combination with 5-FU shows the significant inhibition of tumor growth. This suggests that enhanced activity is achieved between Zt/f2-mediated inhibitory effect and chemoagent-induced cytotoxicity. Third, the inhibitory effect of Zt/f2 is seen in both Balb/c mice with normal immune system and athymic nude mice lacking the T-cell-mediated immunity. Fourth, Zt/f2 treatment alone shows only partial inhibition of tumor growth. In both 3T3-RON160 and HT-29-mediated tumors, only 55% inhibition was observed. Complete tumor remission was not achieved. These results are in line with other anti-RON mAbs such as IMC-41A40 [25]. IMC-41A40 shows tumor inhibition at a range of about 50%. Studies using small molecule kinase inhibitors specific to RON such as compound I also have shown about 50% tumor inhibition [26]. Clearly, additional measures are needed to achieve the maximal therapeutic efficiency to completely block tumor growth. Currently, the mechanisms underlying the Zt/f2 action *in vivo *are unknown. Multiple mechanisms including antibody-mediated immune reactions might be involved in tumor growth inhibition. Nevertheless, data from our current *in vivo *studies suggest that Zt/f2 has potential for targeted cancer therapy. This work should also provide the basis for future development of Zt/f2-based cancer therapy.

## Materials and methods

### Cell Lines and Reagents

NIH3T3 cells expressing RON, RON165, RON160, or RON110 were used as previously described [30]. Human colon cancer HT-29 cells were from ATCC (Manassas, VA). Mouse mAb Zt/g4 to the RONsema domain and rabbit antibody to the RON C-terminal tail were used as previously described [29]. Rabbit IgG cross-reacting with RON from different species was from Santa Crutz Biotechnology (Santa Crutz, CA). Mouse mAb to phosphor-tyrosine, Erk1/2, AKT, and other proteins were from Cell Signaling (Danvers, MA). A peptide containing 49 amino acid coded by exon 11 of the RON gene was synthesized as previously described [16]. Endocytic inhibitor filipin, proteasomal inhibitor lactacystin, and chemoagent 5-FU were from Fisher Scientific (Pittsburgh, PA).

### Generation of hybridoma and purification of mAb

The classical hybridoma method was used to generate mouse mAbs specific to human RON as previously described [29]. NIH-3T3 cells expressing RON or RON160 were used as immunogen to immunize Balb/c mice. Spleen cells from immunized mice were fused with S/p2 myeloma cells. Positive hybridoma lines were established by limiting dilution. Several mAbs such as Zt/f2 and Zt/#1 were purified from individual clones by using protein G Sepharose affinity column.

### Immunoprecipitation and Western blot analysis

These methods were performed using specific antibodies as detailed previously [33]. RON and other proteins were detected by Western blotting using specific antibodies. The membrane also was reprobed with anti-β-actin antibody to ensure the equal sample loading.

### Labeling of mAb with FITC and Immunofluorescent cell surface analysis

The labeling of Zt/f2 with FITC was performed using a FITC labeling kit as previously described [29]. Fluorescent cell surface/cytoplasmic analysis was performed by flow cytometry (FACScan, Becton Dickinson, Franklin Lakes, NJ) as previously described [29]. Cells were treated with specific mAb or control IgG for 45 min followed by goat-anti-mouse IgG coupled with FITC (Jackson Immunolaboratory, Bar Harbor, Maine).

### Cell growth and migration assays

Proliferation of HT-29 cells was determined by counting cell numbers three days after incubation with various mAb as previously described [21]. Cell numbers from individual samples in triplicate were determined by Cellometer Auto-T4 counter (Nexcelon Inc., Lawrence, MA) and expressed as a percentage of cell growth. HT-29 cell migration was determined using a multi-well migration chamber as previously described [28]. Migrated cells were counted and expressed as a percentage of total cells.

### *In vivo *tumor growth and treatment

Induction of tumor growth by 3T3-RON160 cells was performed using Balb/c mice as previously described [21]. HT-29 cell-mediated tumor growth was carried out using athymic nude mice (Taconic, Cranbury, NJ) according to a previously reported method [21]. Briefly, 3T3-RON160 (1 × 10^6 ^cells per mouse) or HT-29 (2 × 10^6 ^cells per mouse) cells were subcutaneously injected into the posterior flank of mice. Three days after inoculation, mice were randomized into different groups. Animals were treated with i.p. injection every three days with Zt/f2, Zt/g4, or control mouse IgG (three mice per group) at a dose of 35 mg/kg. A total of six injections were performed during the duration of the study. Tumors were measured twice each week and tumor volumes were calculated using the formula: 4(*a *x *b*), where *a *was the larger and *b *was the smaller diameter.

For combined treatment, athymic nude mice inoculated with HT-29 cells were used. Treatments began when tumor volume had reached about 9 mm^3 ^(at about day eight). Tumor bearing mice were randomly divided into two groups: G-I and G-II. Mice within these two groups were further randomized to one of the four treatment groups (eight mice per group): PBS control; Zt/f2 treatment (35 mg/kg i.p, every four days for 20 days with a total of five injections); 5-FU treatment (20 mg/kg i.p. for seven continuous days); and Zt/f2 plus 5-FU treatment (combination of Zt/f2 and 5-FU). Mice in G-I were euthanized on day 25 and tumors were weighted and compared among the four groups. Mice in G-II were observed until no survival existed in the PBS group and survival rates were compared among the groups. Differences in mean tumor volume and in mean survival rate were evaluated by Student's *t*-test. The *p *value of comparison (< 0.05) was considered as significant.

## Authors' contributions

HPY and YQZ worked on Zt/f2 production and animal studies; QM, SG, and SSP carried out biochemical studies and receptor internalization experiments. RWZ and MHW designed the study and drafted the manuscript. All authors have read and approved the final manuscript.

## Conflict of interest

The authors declare that they have no competing interests.
